# Improving HIV outcomes in resource-limited countries: the importance of quality indicators

**DOI:** 10.1186/1472-6963-12-427

**Published:** 2012-11-24

**Authors:** Aima A Ahonkhai, Ingrid V Bassett, Timothy G Ferris, Kenneth A Freedberg

**Affiliations:** 1 Divisions of Infectious Disease, Massachusetts General Hospital, 50 Staniford St, 9th Floor, Boston, MA 02114, USA; 2 General Medicine, Massachusetts General Hospital, 50 Staniford St, 9th Floor, Boston, MA 02114, USA; 3 Medical Practice Evaluation Center, Massachusetts General Hospital, 50 Staniford St, 9th Floor, Boston, MA 02114, USA; 4 Partners Healthcare, 50 Staniford St, 9th Floor, Boston, MA 02114, USA; 5 Harvard University Center for AIDS Research, Harvard University, Boston, MA, USA

**Keywords:** HIV/AIDS, Resource-poor, Quality indicator, Quality improvement

## Abstract

**Background:**

Resource-limited countries increasingly depend on quality indicators to improve outcomes within HIV treatment programs, but indicators of program performance suitable for use at the local program level remain underdeveloped.

**Methods:**

Using the existing literature as a guide, we applied standard quality improvement (QI) concepts to the continuum of HIV care from HIV diagnosis, to enrollment and retention in care, and highlighted critical service delivery process steps to identify opportunities for performance indicator development. We then identified existing indicators to measure program performance, citing examples used by pivotal donor agencies, and assessed their feasibility for use in surveying local program performance. Clinical delivery steps without existing performance measures were identified as opportunities for measure development. Using National Quality Forum (NQF) criteria as a guide, we developed measurement concepts suitable for use at the local program level that address existing gaps in program performance assessment.

**Results:**

This analysis of the HIV continuum of care identified seven critical process steps providing numerous opportunities for performance measurement. Analysis of care delivery process steps and the application of NQF criteria identified 24 new measure concepts that are potentially useful for improving operational performance in HIV care at the local level.

**Conclusion:**

An evidence-based set of program-level quality indicators is critical for the improvement of HIV care in resource-limited settings. These performance indicators should be utilized as treatment programs continue to grow.

## Background

### HIV care in low and middle income countries

Since first reported in 1981, HIV/AIDS has claimed the lives of nearly 27 million people worldwide [1,2]. The United Nations Development Program named HIV responsible for “the single greatest reversal in human development” in modern history [3]. In response, the international community has committed to reversing the toll of this pandemic through financial support, political advocacy, and civic engagement [4]. With these efforts, 6.7 million people living with HIV in low-and middle-income countries now have access to lifesaving treatment, representing a 16-fold increase in 7 years [5]. The introduction of antiretroviral therapy (ART) has been estimated to have averted 2.5 million deaths in those settings [5].

This progress has been inconsistent across communities and countries. HIV remains under-diagnosed, many patients present late to care or not at all, some do not receive therapy despite clinical eligibility, and others do not remain in care over time [1,6-12]. Addressing these challenges will require provision of new services in addition to improvement of the quality of existing services. The latter suggests an opportunity to apply the tools of quality improvement (QI) championed in well-resourced nations [4,13].

Several major global HIV donor and advocacy agencies have worked to consolidate performance indicators for HIV programs [1]. Increasing attention has been paid to the quality and impact of HIV services, and to greater alignment with host-country goals. However, several weaknesses remain. The strategic framework for monitoring and evaluation utilized by donor and advocacy agencies may not always be consistent with the improvement model where the focus of measurement is on process and outcome measures, and change is implemented at the smallest replicable unit – the microsystem of healthcare delivery. National and global indicators provide a broad picture of the scope, size, and impact of HIV services, and are important for strategic planning and resource allocation. Therefore, national and global indicators should not be replaced by program-level indicators. However, program-level indicators remain incomplete, and a carefully selected set of measures collected at 6 to 12-month intervals provide important data that can be disseminated to staff to guide QI efforts [1].

Among the available quality indicators for HIV, several critical points including linkage to care, ART eligibility, and ART preparations as well as important patient reported and clinical outcomes are either not represented, or are poorly represented. In addition, current indicator and reporting requirements are often decentralized and dependent on donor agencies [14]. In a study of data quality from the international ART-LINC group, 67% of sites received funding from two sources, and 24% from three [14]. In the same study, only 61% of programs used the same software for data collection and management, creating a gap between measurement, clinical care and improvement, while increasing the cost and complexity of monitoring and evaluation [14]. This study suggests that competing priorities from multiple funding sources create barriers to data collection on additional indicators that may be crucial to local improvement and underscores the importance of identifying a set of measures that will meet reporting, stakeholder, and local quality improvement needs.

The aim of this manuscript is to help care delivery programs better utilize quality improvement methods by providing them with a better ability to assess their own performance. More specifically, we propose a framework for considering performance measures based on the continuum of HIV care. We then identify where gaps exist between available measures and those measure concepts identified by our framework.

To accomplish this aim, we first provide an overview of quality improvement (QI) science in healthcare, highlighting important QI concepts. We then summarize the global HIV-specific QI measurement efforts in resource-limited settings utilized by three pivotal advocacy and donor agencies with broad international reach (UNAIDS, the Global Fund to fight AIDS, Tuberculosis, and Malaria, and the President’s Emergency Plan for AIDS Relief [PEPFAR]). We describe performance along the HIV care continuum, identifying potential foci for quality improvement, and gaps in current measurement efforts. Finally, we propose a set of quality indicators for HIV service delivery programs to address current measurement gaps along the HIV care continuum.

## Methods

To summarize HIV QI concepts and identify existing performance assessment measures, we conducted a literature review using Medline and EMBASE databases in addition to published reports from UNAIDS, the Global Fund, and PEPFAR. Using available literature, we developed an HIV care continuum framework, paying close attention to key process steps and outcomes susceptible to measurement as called for in process improvement theory. Having identified the key process steps required to deliver HIV services, we again searched the literature for studies addressing performance in each of the identified process steps. We also assessed the extent to which existing performance measures addressed process issues identified at each of the process steps. We used the literature to propose additional measures and assessed the extent to which each met National Quality Forum (NQF) recommendations for measure importance, usability and feasibility [15]. Assessment of measurement properties (i.e. validity, reliability, etc.) was beyond the scope of this work.

## Results

### The science of quality improvement in healthcare

Borrowing from industrial quality control methods developed in the 1950’s and 1960’s, healthcare in resource-rich countries has seen rapid growth in the understanding and development of methods that improve quality [16]. Quality improvement relies both on a philosophy, the pursuit of continuous performance improvement, and a diverse armamentarium of methods. These methods include operations research, assessment of healthcare delivery processes, participative management, and setting of performance benchmarks. They also include determining best practices, often codified in practice guidelines, and measuring adherence to those best practices [17].

Central to achieving high quality of care is the definition, observation and measurement of key system indicators – the fundamental metrics in quality improvement measurement. Many healthcare QI experts support the Donabedian model, which suggests that indicators should assess the *structures* (characteristics of physical deployment of resources such as physicians, nurses, buildings and supplies), *processes* (what health professionals do with people), and *outcomes* of care (what happens to people, especially with regard to their health) [18]. These attributes of a system of care are not of equal value. Outcomes matter most, but are also the most challenging to measure. Process measures are a better indicator of healthcare quality than structural measures because they allow assessment of timeliness and appropriateness of care [19]. While individual indicators may reflect specific measures of importance, they may not in isolation lead to an understanding of the broader landscape and affect change that leads to improvement. To address this, the “balanced scorecard” approach was designed to merge a set of measures (including structures, processes and outcomes) across several domains (e.g., timeliness, cost-effectiveness, appropriateness) in a format that would enable managers to translate the organization’s mission into a specific subset of measurable objectives [20].

The development and selection of indicators is linked to data collection, implementation of targeted activities, and evaluation of their impact on health outcomes [16,21]. Once identified, quality indicators should be evaluated for their importance, scientific acceptability, usability, and feasibility [22]. These measures can then be used at the program level (the point of service delivery) to assess healthcare quality and identify areas for improvement. This process is most effectively executed at the level of the microsystem – a small group of people who work together to provide care to patients; who best understand the challenges to improvement; and are responsible for implementing the changes that drive improvement [23]. For HIV treatment programs, such a team may consist of clinicians (physicians, nurses, pharmacists, aides, etc.), counselors, home-based care/outreach workers, and clerical/data entry staff.

### HIV-specific QI measurement in resource-limited countries

The Joint United Nations Program on HIV/AIDS (UNAIDS), along with major funding bodies, has provided guidance and leadership to improve the capacity and quality of HIV care globally. UN member states pledged to regularly report progress on their commitment to reversing the spread of HIV/AIDS; in 2003, the UNAIDS Secretariat developed a set of core indicators to monitor that progress [1,13]. The largest multilateral donor agency for the HIV response, the Global Fund to fight AIDS, Tuberculosis and Malaria (GFATM) was established to increase financing for these infections in 2002 [24]. The US President’s Emergency Plan for AIDS Relief (PEPFAR), the largest bilateral funding organization for the global HIV/AIDS pandemic, was launched by the US Congress in 2003 [25-27].

UNAIDS, the Global Fund, and PEPFAR have adopted an overarching framework for the monitoring and evaluation of national HIV/AIDS programs in host countries. This framework relies on a logic model that measures *inputs, activities, outputs, outcomes,* and *impacts*, which provide information on what goes into a program and the results achieved [28]. The measures are indicators collected at the program, provincial, and local levels, and are aggregated to reflect the progress of national HIV/AIDS programs in meeting country, donor, and advocacy goals. They are multifunctional and provide guidance on the strategic planning, coordination and implementation of programmatic efforts for the global HIV/AIDS response, accountability to donor agencies, and assessments of program effectiveness [28-31].

Despite attempts to align monitoring and evaluation efforts, the UN and others, recognizing a lack of uniformity in key system measures, published a set of 25 core indicators in 2007 on which all countries were to report (Tables 1) [1]. GFATM-supported programs report on a minimum set of 40 national indicators; 25 of these are from the UN General Assembly requirements, and an additional 15 indicators span antiretroviral therapy treatment programs, development of HIV workplace programs, and reduction of stigma (Tables 1) [28]. While some indicators were appropriate for program-level monitoring, many were not, and the Global Fund recognized that additional program-level indicators were needed [28].

**Table 1:** Summary of current HIV testing, treatment, and care indicators

**STEP ALONG CONTINUUM**	**INDICATORS**		
	**UNAIDS**	**Global Fund for HIV, TB, & Malaria**	**PEPFAR**
**a) HIV Testing & Diagnosis**	% high risk persons HIV- tested with known result last 12 months	% high risk persons HIV-tested with known result last 12 months	# persons HIV-tested & received result
	% M & F 15-49y HIV tested with known result last 12 months	% M & F 15-49y HIV-tested with known result last 12 months	% M & F 15-49y HIV-tested with known result last 12 months
		% sexually active M & F 15-24y HIV-tested with known result last 12 months	
**b) Linkage to Care**			
**c) ART Eligibility: Clinical, Laboratory, & Psychosocial Assessment↕**	% patients with incident TB treated for TB & HIV		% patients screened for TB
			% patients that started TB treatment
			# malnourished patients that received therapeutic or supplementary food
			# eligible clients that received food &/or nutrition services
**d) ART Preparation: Literacy Training, OI Prophylaxis, and Adherence Assessment↕**		% eligible adults & children that received co-trimoxazole prophylaxis	# patients that received co-trimoxazole prophylaxis
**e) ART Initiation**	% adults & children with advanced HIV receiving ART	% adults & children with advanced HIV receiving ART	% adults & children with advanced HIV receiving ART
		% ART facilities monitoring CD4 in accordance with national guidelines	# adults and children with HIV infection newly enrolled on ART
		# and% persons starting ART who picked up all ART drugs on time	# adults and children with advanced HIV infection receiving ART
		% ART facilities that have experienced a stock-out of at least one required ART drug in the last 12 months	
**f) Retention in Care**	% adults & children known to be on treatment 12 months after ART initiation		% of adults & children known to be alive & on treatment 12 months after ART initiation
**Clinical Outcomes**			
**Other**		% health facilities that offer ART	# service outlets providing ART services according to national or international standards
			# health workers trained to deliver ART services according to published standards

**Figure 1:**
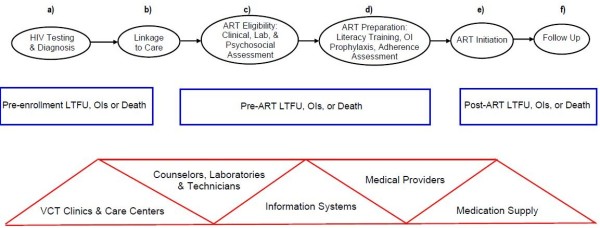
**The Continuum of HIV Care in Resource Limited Settings.** Points A-F represent distinct structures, processes, and outcomes along the HIV care continuum. Triangles (Δ) represent structures, ovals ( ) represent processes, and rectangles (▭) represent outcomes. ART: antiretroviral therapy, LTFU: lost to follow-up, VCT: Voluntary Counseling and Testing, OI: opportunistic infection.

**A-F** are points on the HIV care continuum shown in Figure 1; ART: antiretroviral therapy, TB: tuberculosis; Global Fund recommended indicators, disaggregated by sex, age, and pregnancy status; **↕**reference group is HIV positive patients enrolled in HIV care.

Most recently, PEPFAR changed its monitoring and evaluation framework, calling for increased host country ownership, greater alignment of indicators with host country reporting needs, and inclusion of indicators measuring coverage and quality of services [31]. In addition, since 2006, PEPFAR has implemented a QI initiative, HEALTHQUAL, which helps participants measure key indicators and develop QI programs to meet improvement goals [26]. HEALTHQUAL programs are currently utilized in several PEPFAR countries and many of the indicators endorsed are program-level measures defined to meet program and country needs [26,32].

### The continuum of HIV care as a framework for developing quality improvement measures

Despite improved care resulting from scale-up of the global HIV/AIDS response, substantial morbidity and mortality can still be traced to inadequacies in care delivery at distinct points along the HIV care continuum (Figure 1). These critical points include diagnosis, linkage to care, initiation of antiretroviral therapy, treatment or prophylaxis of opportunistic infections, and retention in care over time [8,33]. Successful patient outcomes are contingent upon a high degree of success at each and every point on this continuum [34,35]. While barriers to optimal care are not yet fully elucidated, quality indicators focused on essential points along the care continuum will be necessary to identify weaknesses and design interventions to maximize performance. In the descriptions below we highlight the seven key process steps in the HIV care continuum; to: 1) summarize the evidence regarding performance at each step, and 2) present our assessment of the opportunities for performance measure development.

#### a. HIV testing and diagnosis

The entry point to HIV treatment is through testing and diagnosis, yet only 30% of women and 17% of men in Sub-Saharan Africa are aware of their HIV status [4]. Historically, HIV testing has been provider-driven and prompted by symptoms manifested in the advanced stages of HIV disease [36]. Further, uptake of voluntary counseling and testing (VCT) at health service sites has been estimated at under 50% in several Sub-Saharan African countries [37,38]. Recognizing the importance of expanding HIV testing, the WHO recommended provider-initiated, opt-out HIV testing in 2007 [39]. In addition to health service-based VCT, other models, including free-standing, mobile, community-based, home-based, and self-testing have also been studied [36,37,39-42]. Routine monitoring of VCT uptake may inform the most successful models.

Quality measures for HIV/AIDS treatment programs should first assess how the system performs with regard to HIV testing. The current UNAIDS, PEPFAR, and Global Fund reports contain indicators that measure HIV testing in several sub-populations [1]. Given the poor uptake of most VCT models to date, additional indicators highlighting the adoption of alternative VCT models (such as routine, healthcare-based, or home-based) could be used to provide benchmarks for future performance [36].

#### b. linkage to care

After diagnosis, HIV-infected patients must be successfully linked to treatment programs. There is a paucity of data on linkage to care in resource-limited countries. Studies in southern Africa suggest that 50-70% of patients who tested positive for HIV enrolled in clinical care within 3–9 months of diagnosis, representing the largest lost opportunity to engage patients along the HIV care continuum [6,7,43]. In one systematic review of HIV treatment programs in Sub-Saharan Africa a median of 59% of patients successfully link from HIV testing to CD4 testing or clinical staging [44]. Features such as multiple care sites (e.g. for HIV testing, CD4 count testing, and tuberculosis treatment), long wait times for appointments and receipt of test results, in addition to transportation barriers and medication costs are obstacles to effective linkage [8,10,45,46]. Definitions of linkage to care are not straightforward [44]. In some clinical settings, patients may report to a facility outside of a clinic for CD4 testing, and thus may obtain CD4 testing without being linked to care in an HIV treatment program. In addition, some have argued that patients who are not yet eligible for ART at the time of HIV diagnosis may need to have multiple clinical visits to demonstrate effective linkage to care [44,46].

While the barriers to linkage to care are not fully understood, indicators that assess delays in these steps, such as time from diagnosis to enrollment in care can still be defined [6,7]. Such measures are currently lacking, and are challenging to programs that do not have health system infrastructure that supports patient identification across different healthcare providers. Nonetheless, measures for linkage to care must identify patients who have enrolled in HIV clinics. A recent systematic review of retention in HIV care from testing to treatment in Sub-Saharan Africa calls for standardization of terms to facilitate the study of linkage to care across multiple care sites. The authors define 2 stages addressing care linkage. Stage 1 begins after receipt of a positive HIV test, and ends with the receipt of CD4 count or clinical staging result and referral to ART or pre-ART care; stage 2 begins after referral to pre-ART care, and ends with ART eligibility [44]. Adoption of such standard definitions would be an important step in developing quality measures.

#### c. Antiretroviral therapy eligibility

Once enrolled in HIV care, patients should be staged with clinical and psychosocial evaluation in addition to CD4 count testing to assess eligibility for antiretroviral therapy, and to screen and prophylax for opportunistic infections [47]. Given evidence that starting treatment earlier reduces the risk of AIDS and death, the WHO in 2009 increased the recommended threshold for ART initiation from <200 cells/uL to <350 cells/uL [48]. Nonetheless, patients in most low-income countries present to care much later (median CD4 108 cells/uL) than their counterparts in high-income countries (median 234 cells/uL) [11]. This late presentation is clearly associated with an increased risk of death [11,48]. Further, recent evidence from HPTN 052, a trial of antiretroviral therapy in couples with discordant HIV status, showed significantly less transmission when antiretroviral therapy was initiated with a CD4 cell count of 350–500 cells/uL compared to less than 250 cells/uL. Thus, it is likely that antiretroviral therapy eligibility will move even earlier in disease, and will present new challenges to LMIC [49].

Tuberculosis (TB) is the leading cause of illness and death among people living with HIV worldwide [50]. Screening for TB upon entry into HIV clinic, prompt treatment for active TB, and disease prophylaxis were recommended by the WHO as priority interventions in 2010, but uptake is poor [50]. In 2009, only 5% of the 33 million people living with HIV were screened for TB, and optimal screening methods are yet to be established [51-54].

Though not standard practice, increasing programmatic support has also focused on food supplementation, given the importance of food insecurity and malnutrition on outcomes of HIV-infected patients [55-57]. Recent data from resource-limited settings demonstrates that food assistance is associated with increased body mass index and better clinic adherence [58,59]. In addition, one study from rural Uganda suggests that early initiation of antiretroviral therapy may improve physical health status and thereby improve food security [60].

#### d. Antiretroviral therapy – preparation and initiation

After disease staging, most HIV treatment programs have incorporated a process to assess antiretroviral therapy readiness that includes psychosocial assessment, adherence counseling, and identification of a treatment supporter. This process typically spans several clinical visits [61-63]. Many programs describe long delays in antiretroviral therapy initiation, with patients waiting up to 120 days to complete adherence training [8]. Stock-outs of essential drugs affect at least 11% of patients on antiretroviral therapy treatment, according to one study in Côte d’Ivoire. Resultant treatment discontinuation is associated with increased risk of care interruption or death [64]. Though poor adherence to antiretroviral therapy is associated with increased mortality, the optimal format and duration of antiretroviral therapy literacy training, and its impact on adherence, have not been established [65]. One study in Uganda found that completion of adherence training prior to ART initiation did not improve adherence [66]. In addition to these delays, 16-45% of patients are also lost from care before starting antiretroviral therapy, and others die while waiting to start treatment [8,44,46,67].

Substantial delays also exist in the time from enrollment in care to full clinical assessment and ART initiation [6,7,61]. Potential indicators could measure the percentage of patients enrolled in an HIV care program that receive a CD4 count within 3 months of HIV diagnosis, or the delay to initiation of ART in eligible patients. Indicators could also be defined to determine the proportion of patients screened for concomitant TB infection, which is a critical problem in those who are HIV-infected.

#### e. Retention in care

For patients enrolled in antiretroviral therapy programs, high program attrition rates have been described, with 15-55% of patients lost from care between 6 and 36 months [9,10,45]. In programs that have actively traced patients lost to follow-up, up to 40% were found to be dead [8,10,63]. Some patients transfer care to other programs, and others cycle in and out of care with brief interruptions but worse virologic control than those who remain in care [68,69]. As a result, patient retention has been increasingly recognized as an important program indicator.

Once initiated on antiretroviral therapy, patients should be monitored for medication adherence and toxicity in accordance with local guidelines. These efforts require reliable medication supply chains and laboratory services, which can be assessed through specific indicators such as the number of stock-outs of essential medications per 6-month period. These measures would identify the greatest delays in the system, potential interventions, and appropriate targets for future performance.

#### f. Clinical outcomes

Optimally, HIV-infected patients will remain in care, on antiretroviral therapy, with controlled disease. However, patients in resource-limited settings who are initiated on antiretroviral therapy are 3.5 times more likely to die than patients in resource-rich countries [11]. This risk of death is highest in the first few months after antiretroviral therapy initiation, and has been attributed to presentation to care with advanced disease [12,70]. Despite the increased risk of early death, patients in resource-limited and resource-rich settings appear to gain similar immunologic and virologic benefit from antiretroviral therapy [71-73]. Approximately 70% of those on therapy achieve virologic suppression at 6 months [11,72]. WHO has provided guidelines for treatment failure based on CD4 count response, but discordant virologic and immunologic responses to antiretroviral therapy may occur in up to 20-30% of patients, and viral load monitoring is not widely available in many resource-limited settings [47,74,75]. Patients who do fail therapy may require intensive adherence interventions, or switching to more costly second and third-line treatment regimens [76].

Improved data collection mechanisms, unique patient identifiers, electronic medical records, use of patient trackers, and program cost subsidies may reduce loss to follow-up [10,14,45]. UNAIDS and PEPFAR define one indicator that measures 12-month retention rates, but the complementary measure, loss to follow-up is less well defined [77]. Geng *et al*. suggest a sample-based approach to estimate the outcomes of these patients, which may be appropriate to help programs understand barriers to retention [78]. In addition to patients initiated on antiretroviral therapy, it is important to monitor patients who are antiretroviral therapy ineligible at the time of enrollment, to ensure that they are not lost to follow-up despite earlier presentation to care. An indicator measuring the percentage of patients antiretroviral therapy ineligible at baseline who receive a follow-up CD4 count in 6 months could capture this.

An indicator scorecard for HIV programs in resource-limited settings should ultimately contain metrics reflecting clinical and patient-reported outcomes. These should be important measures in their own right, or have known association with clinical outcomes, such as retention in care, mortality (both before and after antiretroviral therapy initiation), as well as treatment failure and switches to second-line therapy*.*

#### g. Patient-reported outcomes: health-related quality of life and patient satisfaction

The Institute of Medicine defines patient-centered care (care that is respectful and responsive to individuals) as one of the six aims for improving the delivery of quality health care [79]. Patient-reported outcomes such as quality of life and service satisfaction are examples of measures that characterize patient-centered care [80]. Health related quality of life (HR-QOL) has been increasingly recognized as an important outcome, particularly as HIV has transformed into a chronic disease in the effective ART era [81,82]. HR-QOL is a complex measure that includes several dimensions including physical function, symptoms, performance of social roles, emotional status, cognitive functioning, and individual feelings about health [80,83,84]. Several HR-QOL scales have been developed for use across international settings (WHO-QOL, PRO-QOL) and for HIV research in research-rich settings (MOS-HIV, FAHI, HOPES, HAT QOL, AIDS-HAQ, MQOL-HIV) [83-90]. Most of these instruments were developed in the pre-ART or pre-effective ART era, are long, and were developed for research rather than routine clinical practice. There is no consensus on the optimal tool for measuring HR-QOL among patients infected with HIV [81].

Several studies have described a range of challenges for poor patients seeking medical care in resource-limited settings and across the globe, such as difficulty accessing care, high direct and indirect costs of care, long wait times, and poor treatment by staff [10,45,78,91-94]. These challenges can all influence patient satisfaction, defined as the extent to which a patient’s health care experience matches his or her expectations [95]. There has been relatively little focus on patient satisfaction with HIV care services in resource-limited settings [96-101].

More effort is needed to identify and standardize metrics for quality of life and care satisfaction that can be used in routine clinical practice in resource-limited settings [84]. The AIDS Clinical Trials Group (ACTG) has used a brief 2-question measure (one 5-point likert scale, and another rating on a scale of 1–100) to evaluate patient reported health status in the ACTG Longitudinal Linked Randomized Trials protocol [102]. Health status measures a dimension of quality of life that is impacted by health care satisfaction [83-85]. These ACTG measures may provide a useful starting point for the assessment of important patient reported-outcomes to promote patient-centered care. Because of the constraints posed by limited resources, it becomes even more critical to focus on indicators that will have the greatest impact on each health system and the patients it serves [14].

### Proposed quality measures for HIV programs in resource-limited countries

The set of quality indicators currently used to assess the treatment and care of HIV programs in resource-limited countries lacks focus on several areas most likely to improve HIV outcomes (Table 1) [11,77,103]. The tension between funder needs for accountability and strategic planning, and evaluation and program needs for local improvement, may be difficult to harmonize with a single set of indicators. Therefore, using the model in Figure 1 as a framework, we assessed the literature describing the operational challenges facing HIV care in resource-poor settings. Utilization of this framework, along with the selection of indicators measuring both processes of care and key clinical and patient reported outcomes guided the development of a set of 24 program-level measure concepts to guide improvement for HIV treatment programs (Table 2). The measure concepts were designed to address the limitations of current indicators described above, as well as Utilization of this framework, along with Utilization of this framework, along with the NQF criteria of importance, usability, and feasibility*.*

**Table 2:** Proposed scorecard of program-level quality measures

**STEP ALONG CONTINUUM**	**MEASURE**
**a) HIV Testing and Diagnosis**	▪% of patients diagnosed on site **[P]**
	▪% of patients diagnosed in other medical facilities **[P]**
	▪% of patients diagnosed via home-based test **[P]**
	▪% of patients diagnosed in mobile testing unit **[P]**
	▪% of couples whose partners have been HIV tested and are aware of results **[P]**
**b) Linkage to Care**	▪ Median days from HIV diagnosis to referral for ART or pre-ART care **[P]**
	▪% of patients ART *in*eligible at baseline who receive a follow-up CD4 count in 6 months **[P]**
	▪ Median days from clinic enrollment to ART eligibility **[P]**
	▪% of patients who are enrolled in HIV clinic, received CD4 count & results within 3 months of HIV diagnosis **[P]**
	▪% of patients with CD4 count ≤ 200 cells/uL, & ≤ 350 cells/uL at presentation **[P]**
**c) ART Eligibility: Clinical, Laboratory & Psychosocial Assessment**	▪% of patients screened for tuberculosis **[P]**
	▪% of eligible patients provided with nutritional supplementation **[P]**
	▪% of ART-eligible patients who died before ART initiation **[O]**
**d) ART Preparation: Literacy Training, OI Prophylaxis, and Adherence Assessment**	▪ Median days from enrollment to ART initiation for eligible patients **[P]**
	▪ Median days from enrollment to completion of ART literacy training for eligible patients **[P]**
**e) ART Initiation**	▪ # drug stock outs in last quarter for first-line ART drugs or cotrimoxazole **[P]**
	▪ Local guideline concordance (e.g. CD4 testing, adherence monitoring, assessment for drug toxicity, OI screening & prophylaxis) **[P]**
**f) Retention in Care**	▪% of patients retained in care 6 and 12 months from enrollment (ART eligible and ART-ineligible) **[O]**
	▪% of patients who transferred care to other clinics 6 and 12 months from enrollment **[O]**
	▪% of patients deemed lost to follow-up who have been contacted by clinic staff to determine outcome **[O]**
**g) Clinical Outcomes**	▪% of patients on ART with undetectable viral load at 12 months **[O]**
	▪% of patients on ART requiring switch to second-line therapy for treatment failure at 12 and 24 months **[O]**
	▪% of patients (ART-eligible on and off ART) who died 12 months after enrollment **[O]**
**h) Patient Reported Outcomes**	▪ patient-reported health status 6 and 12 months after clinic enrollment **[O]**

[P] = process measure, [O] = outcome measure.

To assess HIV testing and diagnosis, we propose five process measures to illustrate where patients who successfully present to clinic receive their testing (clinic-based vs. home or mobile testing), and to inform whether partners of known HIV-infected patients are receiving testing. These data will allow HIV treatment programs to provide critical feedback about which local HIV testing modalities are most successful and may benefit from scale-up, in addition to which are least successful and could benefit from improvement efforts.

To determine program performance around linkage to HIV care, we recommend five process measures measuring both stage 1 (from receipt of a positive HIV test to receipt of CD4 count or clinical staging result and referral to ART or pre-ART care) and stage 2 (from referral to pre-ART care to ART eligibility) linkage as defined by Rosen et al., [44]. The most effective measurements of linkage to care require the merging of data from HIV testing and treatment centers, which are rarely available in resource-limited settings [44,46]. Our proposed measures can be captured at the point of HIV care alone.

We propose six process and one outcome measure to assess program performance on it’s efficiency in determining ART eligibility and effectively initiating patients on ART. These include screening for opportunistic infections and nutritional needs in addition to ensuring adequate drug supply, measuring the time it takes to identify and initiate ART-eligible patients on life-saving therapy, and concordance with local ART monitoring guidelines. The proposed outcome measure provides programs with important data on potentially preventable deaths of ART-eligible patients who die before starting therapy.

Finally, we outline five outcome and one process measure addressing retention in care, patient health status, and important clinical outcomes (virologic suppression, use of second-line therapy, and mortality). Our measures of care retention, in addition to assessing this as an outcome, also necessitate that HIV care delivery programs ask whether they have intervened to decrease LTFU and improve retention. This set of measures encompasses data on endpoints important to patients, clinicians, and administrators.

## Discussion

Our proposed measurement framework and set of program-level quality indicators have several strengths. The HIV care continuum is an increasingly utilized paradigm for implementation practice and research in HIV service delivery, and thus anchors quality measurement around a familiar construct for service providers. Building our framework around the HIV care continuum also allows us to take advantage of implementation research identifying weaknesses in the care continuum, and potential areas for quality improvement. Our identification of measures reflecting processes and outcomes of care moves away from volumetric endpoints that highlight quantity rather than quality of services. Finally, standardization of a set a quality measures allows for comparison of performance across programs, enabling the formulation of benchmarks for appropriate care and identifying best practices in care delivery.

Taken in combination, the proposed measures form the basis of a balanced scorecard of crucial, evidence-based objectives for successful HIV service delivery programs in resource-limited settings. The proposed measure concepts will require further specification and standardization before they can be implemented as indicators (as described above). As is the case in the development of performance measures, some will prove more useful than others; this is difficult to predict prior to implementation. Because program strengths and weaknesses differ from site to site, some programs will find certain measures more useful than others.

These proposed measures also have limitations. The NQF suggests that quality measures meet 4 criteria: importance, usability, feasibility, and scientific acceptability. While we considered the NQF’s criteria for performance measures (importance, usability, and feasibility), in making recommendations, the latter two components can only be fully appreciated once measures are specified and field-tested. Usability and feasibility will invariably depend upon the data collection systems available at the program level. An assessment of scientific acceptability (reliability and validity) of the proposed measures will also be important. Finally, measurement alone will not improve the quality of patient care without being linked explicitly to the dissemination of data to care teams, and empowering these teams to adapt care processes to improve performance and the quality of care provided. Building this type of approach to quality improvement into organizations in well-resourced countries has proved challenging, so there will undoubtedly be challenges in resource-limited settings as well.

## Conclusions

An aphorism in quality improvement science ascribed to one of its founders, William Deming, states; “*Every system is perfectly designed to achieve exactly the results it gets.*” With the rapid scale-up of life-saving therapy for HIV/AIDS, it is important to consider the quality challenges in the system that has emerged for this care in resource-limited settings. The challenges are highlighted by inadequate HIV testing and late presentation to care, poor linkage, major delays in ART initiation, and high loss to follow-up rates, all of which limit the life-saving gains that the system aims to promote. To improve the quality of care provided, identification and measurement of a quality indicator scorecard that reflects important processes and outcomes along the continuum of HIV care is critical. There is now substantial global commitment to improving the quality of HIV care in resource-limited settings. Focused efforts to define and improve upon the most important quality indicators for HIV-infected individuals are crucial as programs continue to grow.

## Abbreviations

(QI): Quality Improvement; (ART): Antiretroviral therapy; (UNAIDS): United Nations Program on HIV/AIDS; (GFATM): Global Fund to fight AIDS Tuberculosis and Malaria; (PEPFAR): President’s Emergency Plan for AIDS Relief; (VCT): Voluntary Counseling and Testing; (ACTG): AIDS Clinical Trials Group.

## Competing interests

The authors declare that they no competing interests.

## Authors’ contributions

AA and KAF conceived of the argument. AA drafted the initial manuscript. IVB and TGF refined the argument. IVB, TGF, and KAF and provided critical revisions to the manuscript. All authors read and approved the final manuscript.

This work was supported in part by National Institutes of Allergy and Infectious Disease (T32 AI 007433, K23 AI 068458, R01 AI058736) and the National Institute of Mental Health (R01 MH090326).

## Pre-publication history

The pre-publication history for this paper can be accessed here:

http://www.biomedcentral.com/1472-6963/12/427/prepub
